# Checkpoint Inhibitor-Associated Autoimmune Diabetes Mellitus Is Characterized by C-peptide Loss and Pancreatic Atrophy

**DOI:** 10.1210/clinem/dgad685

**Published:** 2023-11-24

**Authors:** Linda Wu, Matteo Salvatore Carlino, David Alexander Brown, Georgina Venetia Long, Roderick Clifton-Bligh, Rhiannon Mellor, Krystal Moore, Sarah Christina Sasson, Alexander Maxwell Menzies, Venessa Tsang, Jenny Elizabeth Gunton

**Affiliations:** Westmead Institute for Medical Research, Centre for Diabetes and Endocrinology, Westmead 2145, NSW, Australia; Department of Endocrinology, Westmead Hospital, Westmead 2145, NSW, Australia; Faculty of Medicine and Health, The University of Sydney, Camperdown 2050, NSW, Australia; Melanoma Institute Australia, The University of Sydney, Wollstonecraft 2065, NSW, Australia; Faculty of Medicine and Health, The University of Sydney, Camperdown 2050, NSW, Australia; Melanoma Institute Australia, The University of Sydney, Wollstonecraft 2065, NSW, Australia; Department of Oncology, Westmead Hospital, Westmead 2145, NSW Australia; Westmead Institute for Medical Research, Centre for Diabetes and Endocrinology, Westmead 2145, NSW, Australia; Faculty of Medicine and Health, The University of Sydney, Camperdown 2050, NSW, Australia; Institute of Clinical Pathology and Medical Research, Department of Immunology, NSW Health Pathology, Westmead 2145, NSW, Australia; Department of of Immunology, Westmead Hospital, Westmead 2145, NSW Australia; Faculty of Medicine and Health, The University of Sydney, Camperdown 2050, NSW, Australia; Department of Medical Oncology, Royal North Shore Hospital, St Leonards 2065, NSW, Australia; Faculty of Medicine and Health, The University of Sydney, Camperdown 2050, NSW, Australia; Department of Endocrinology, Royal North Shore Hospital, St Leonards 2065, NSW, Australia; Department of Oncology, Westmead Hospital, Westmead 2145, NSW Australia; Department of Radiology, Westmead Hospital, Westmead 2145, NSW Australia; Faculty of Medicine and Health, The University of Sydney, Camperdown 2050, NSW, Australia; Department of of Immunology, Westmead Hospital, Westmead 2145, NSW Australia; Faculty of Medicine and Health, The University of Sydney, Camperdown 2050, NSW, Australia; Department of Medical Oncology, Royal North Shore Hospital, St Leonards 2065, NSW, Australia; Faculty of Medicine and Health, The University of Sydney, Camperdown 2050, NSW, Australia; Department of Endocrinology, Royal North Shore Hospital, St Leonards 2065, NSW, Australia; Westmead Institute for Medical Research, Centre for Diabetes and Endocrinology, Westmead 2145, NSW, Australia; Department of Endocrinology, Westmead Hospital, Westmead 2145, NSW, Australia; Faculty of Medicine and Health, The University of Sydney, Camperdown 2050, NSW, Australia

**Keywords:** checkpoint inhibitors, diabetes, type 1 diabetes, autoimmune diabetes, immune-related adverse events

## Abstract

**Objective:**

To conduct a multicenter case series characterizing the clinical characteristics at presentation and pancreatic volume changes of patients with checkpoint inhibitor-associated autoimmune diabetes (CIADM).

**Research Design and Methods:**

Electronic medical records were reviewed with 36 consecutive patients identified with CIADM, as defined by (1) previous immune checkpoint inhibitor (ICI) therapy, (2) new-onset hyperglycemia (blood glucose level ≥ 11.1 mmol/L and/or glycosylated hemoglobin ≥ 6.5%), and (3) insulin deficiency [C-peptide <0.4 nmol/L or diabetic ketoacidosis (DKA)] within 1 month of presentation. Pancreatic volume was available and measured using computed tomography volumetry for 17 patients with CIADM and 3 sets of control patients: 7 with ICI-related pancreatitis, 13 with asymptomatic ICI-related lipase elevation, and 11 ICI-treated controls for comparison.

**Results:**

All patients had either anti-programmed cell death protein 1 or anti-programmed cell death ligand 1 therapy. Median time from ICI commencement to CIADM diagnosis was 15 weeks. At presentation, 25 (69%) had DKA, 27 (84%) had low C-peptide, and, by 1 month, 100% had low C-peptide. Traditional type 1 diabetes autoantibodies were positive in 15/35 (43%). Lipase was elevated in 13/27 (48%) at presentation. In 4 patients with longitudinal lipase testing, elevated levels peaked 1 month prior to CIADM diagnosis. Pancreatic volume was lower pre-ICI in CIADM patients compared with controls and demonstrated a mean decline of 41% from pretreatment to 6 months post-CIADM diagnosis.

**Conclusion:**

Pronounced biochemical and radiologic changes occur during CIADM pathogenesis. Rapid loss of C-peptide is a distinct characteristic that can be used to aid diagnosis as autoantibodies are often negative.

In recent years, immune checkpoint inhibitors (ICIs) have not only transformed the landscape of oncological therapy but also enhanced our understanding of mechanisms that can result in autoimmunity. ICIs block critical immune checkpoints such as programmed cell death protein 1 (PD-1) and cytotoxic T-cell-associated antigen 4 (CTLA-4). These agents show significant anti-tumor immune efficacy in a range of malignancies including melanoma and non-small cell lung cancer but also carry a risk of triggering novel autoimmune toxicities known as immune-related adverse events (irAEs) (1–3).

Checkpoint inhibitor-associated autoimmune diabetes mellitus (CIADM), also known as immune checkpoint inhibitor-induced diabetes mellitus, is an increasingly prevalent novel form of diabetes occurring subsequent to ICI therapy with presumed autoimmune pancreatic β-cell destruction. CIADM is a relatively uncommon irAE with an incidence of 0.2% to 1.4% (4–9). However, when considered in the context of 43.6% of all cancer patients in the United States being eligible for ICI therapy (10), this translates to thousands of new CIADM patients per year in the United States alone.

There is a lack of consensus on the diagnostic criteria for CIADM, and the reporting of phenotypical data such as type 1 diabetes (T1D) autoantibodies, serum C-peptide, and exocrine pancreatic inflammation has been variable. In a previous meta-analysis of all reported CIADM cases conducted by our group (11), we demonstrated that low C-peptide is a universal feature of CIADM that correlates with a lifelong insulin dependence, whereas T1D autoantibodies are less reliable. On these grounds we have applied our proposed diagnostic criteria to our case series, which includes (1) new-onset hyperglycaemia and (2) evidence of insulin deficiency with rapid loss of C-peptide at presentation or by 1 month post-diagnosis.

It has been suggested in small case series that pancreatic volume rapidly reduces during the course of progression to overt CIADM (12, 13). No previous comparisons have been made to patients on ICIs alone or patients with pancreatic irAEs but without diabetes. Furthermore, measures of exocrine pancreatic disease such as lipase or fecal elastase have not been extensively analyzed.

While awareness of CIADM is growing, there remain large knowledge gaps in our understanding of the condition. In particular, it is unclear what factors may predict CIADM development, and there is yet to be a clear consensus on diagnostic criteria. We conducted a multicenter case series characterizing the typical presentation of patients with CIADM, as well as the associated exocrine pancreatic changes during the course of disease pathogenesis.

## Research Design and Methods

Patients with new-onset diabetes after ICI treatment were identified during the period January 2015 to March 2023 from 4 quaternary oncological facilities in Sydney, Australia. Human Research Ethics approval was obtained for review of electronic medical records in a multicenter retrospective study (2021/PID00003). Ten patients from this cohort had previously been published (6), although data on further clinical parameters including lipase, glucose, and pancreatic volumetry have been obtained and analyzed.

Patients were included as having a diagnosis of CIADM if they met the following criteria detailed in our recent meta-analysis (11): (1) previous ICI exposure and (2) new-onset hyperglycemia [blood glucose level ≥ 11.1 mmol/L and/or glycosylated hemoglobin; (HbA1c) ≥ 6.5%] and (3) evidence of insulin deficiency [C-peptide <0.4 nmol/L or diabetic ketoacidosis (DKA)] within 1 month of presentation. Patient demographics, biochemistry, oncological characteristics, antibody status, and human leukocyte antigens (HLA) haplotyping was recorded. Antibodies were tested via hospital laboratory using RSR® antiglutamic acid decarboxylase antibodies ELISA (RSR Limited, catalog no. GDE/96, RRID:AB_2910239), RSR® anti-IA-2 ELISA (RSR Limited, catalog no. IAE/96/2, RRID:AB_2910240), RSR® anti-insulin antibody radioimmunoassay (RSR Limited, catalog no. IAA/100, RRID:AB_3073785), and EuroImmun anti-ZnT8 ELISA (EUROIMMUN, catalog no. EA 1027-9601, RRID:AB_3073786). HLA types were defined as “susceptible,” “protective,” or “not associated” based on previous studies in patients with type 1 diabetes (14). A complete case analysis approach was used to address missing data, and patients with inadequate data to confirm a diagnosis of CIADM were excluded.

Pancreatic volumetry was performed using Vitrea® software (Tochigi, Japan). Volume was calculated using the in-built summation of area technique as previously published (15), with pancreas parenchyma manually outlined on each section. Computed tomography (CT) scans were obtained for 3 groups of patients: (1) patients with CIADM, (2) patients treated with ICIs who developed autoimmune pancreatitis, (3) patients treated with ICIs who developed asymptomatic lipase elevation, and (4) ICI-treated controls with normal lipase values. Control patient groups were obtained through a retrospective review of the ICI-treated patient database at Melanoma Institute Australia with diagnoses as recorded on medical records by the treating physician (Royal Prince Alfred Hospital Research Ethics Committee Protocol No. X10-0305 and HREC/10/RPAH). For each group, CTs were obtained from time points prior to ICI commencement; while on ICI therapy (timepoint >6 months on therapy); and at time of diagnosis of CIADM, pancreatitis, or lipase elevation and 6 months post-diagnosis, if available.

R software was used for statistical analysis (version 4.1.1). Normal distribution was tested with Shapiro-Wilk testing. One-way ANOVA analysis was performed to determine the significance of the volumetry changes across time periods and detect differences between patient groups. Difference in time to CIADM between groups was compared via log-rank testing. Student's t-test and chi-square testing were used to compare between groups for continuous and categorical variables, respectively, for parametric variables and Wilcoxon signed rank test for nonparametric variables.

## Results

Thirty-six patients met the inclusion criteria for CIADM. Median age at diagnosis was 64 years, and 58% of patients were male (n = 21). Mean baseline body mass index was 24.7 kg/m^2^, and 14% had pre-existing type 2 diabetes (n = 5), requiring diet control alone in 2 patients and oral hypoglycaemic agents in 3.

Melanoma was the most common malignancy (n = 23, 64%), followed by non-small cell lung cancer (n = 7, 19%). All patients were exposed to either anti-programmed cell death protein 1 (PD1) or anti-programmed cell death protein ligand 1 (PDL1) class ICIs, with 51% receiving combination anti-PD1 and anti-CTLA-4 therapy, 43% anti-PD1 monotherapy, and 6% anti-PDL1 monotherapy. Many patients (78%, n = 28) experienced other irAEs; the most common were thyroid disease (42%, n = 15), hepatitis (14%, n = 5), hypophysitis (14%, n = 5), pancreatitis (11%, n = 4), and colitis (8%, n = 3).

**Figure 1.**
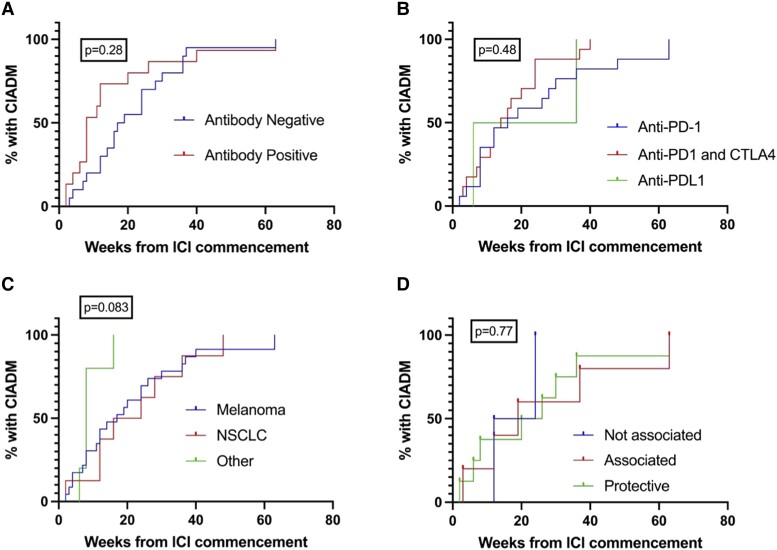
Cumulative incidence curves from ICI exposure to development of CIADM. Cumulative incidence curves are stratified by (A) autoantibody positivity; (B) ICI type; (C) type of malignancy; (D) results of HLA typing for T1D susceptibility. Abbreviations: CIADM, checkpoint inhibitor-associated autoimmune diabetes; CTLA-4, cytotoxic T-cell-associated antigen 4; HLA, human leukocyte antigens; ICI, immune checkpoint inhibitor; NSCLC, non-small cell lung cancer; PD-1, programmed cell death protein 1; PD-L1, programmed cell death ligand 1; T1D, type 1 diabetes.

Median time from the start of ICI to diagnosis with CIADM was 15 weeks (interquartile range 8–26.5 weeks). Figure 1 depicts the time to diabetes diagnosis from initial exposure to immunotherapy, stratified by autoantibody status at diagnosis of CIADM, type of ICI, malignancy, and HLA typing if performed. These factors were not found to be significantly associated with a difference in time to diagnosis.

Biochemistry and autoantibodies at presentation are summarized in Table 1. Of the 14 patients tested, 8 (57%) had HLA susceptibility haplotypes for T1D and 2 (15%) had protective haplotypes. No significant difference in C-peptide values or DKA incidence was found between groups based on subanalyses stratified by HLA susceptibility, cancer type, ICI type, or lipase levels.

**Table 1.** Biochemistry and autoantibodies at presentation with CIADM

		n (%)
DKA at presentation		25 (69)
DKA/HHS overlap at presentation		6 (17)
Mean glucose (mmol/L) ± SD		34.5 ± 15.1
Mean ketones (mmol/L) ± SD		4.3 ± 3.0
Mean C-peptide (nmol/L) ± SD		0.21 ± 0.19
Low C-peptide (<0.4 nmol/L)		27/32 (84)
HbA1c (%)		8.2 ± 1.7
Autoantibody positive	Any	15 (43)
	Anti-GAD (n = 35)	15 (43)
	Anti-IA2 (n = 35)	1 (3)
	Anti-ZnT8 (n = 35)	2 (6)
	Anti-insulin (n = 8)	0 (0)
	Antibody negative	20 (57)
Elevated lipase (normal range <60 u/L, n = 27)		13 (48)
Low fecal elastase (normal range <200 ug/g, n = 10)		9 (90)

Abbreviations: anti-GAD, antiglutamic acid decarboxylase antibodies; anti-ICA, anti-islet cell antigen antibodies; anti-ZnT8, anti-Zinc transporter 8 antibodies; CIADM, checkpoint inhibitor-associated autoimmune diabetes; DKA, diabetic ketoacidosis; HbA1c, glycosylated hemoglobin; HHS, hyperosmolar hyperglycemic state.

**Figure 2.**
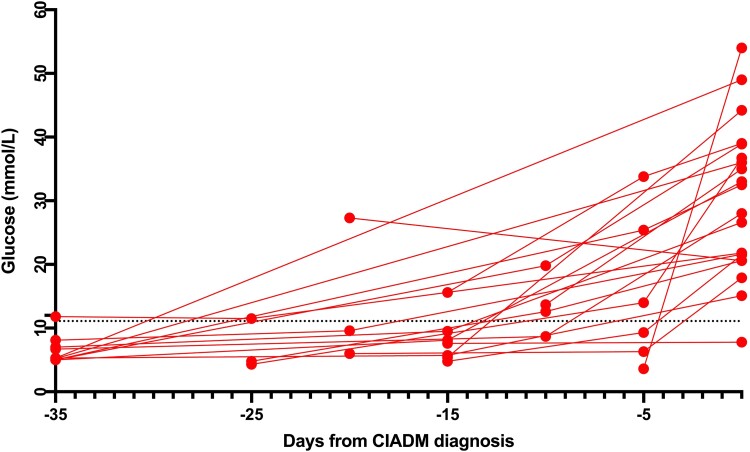
Serum glucose prior to CIADM diagnosis (n = 20). The dotted black line shows the cut-off (≥11.1 mmol/L) for a diabetic-range random glucose. Abbreviations: CIADM, checkpoint inhibitor-associated autoimmune diabetes.

Figure 2 depicts serum glucose levels detected on routine biochemistry prior to formal CIADM diagnosis for the 20 patients who had glucose measurements preceding diagnosis. Eight of 20 patients had an abnormal random glucose level meeting criteria for diabetes (≧11.1 mmol/L) preceding CIADM diagnosis.

## Serum Lipase and Pancreatic Exocrine Function

**Figure 3.**
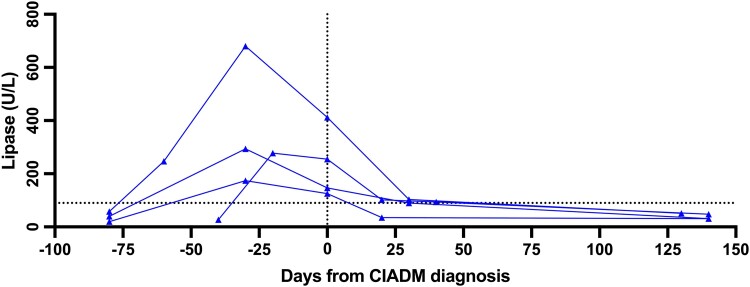
Lipase level during time of diagnosis for CIADM patients (n = 4). Dotted line represents upper limit normal range (60 U/L). Abbreviations: CIADM, checkpoint inhibitor-associated autoimmune diabetes.

Of the 27 patients tested for lipase, values were elevated in 13 (48%), and 4 patients had overt ICI-related pancreatitis. Figure 3 depicts lipase levels at the time of presentation for patients who had elevated lipase and had serial measurements (n = 4), showing a distinct rise in lipase that precedes the onset of CIADM by roughly 1 month. Ten patients had fecal elastase performed to further evaluate the extent of pancreatic insufficiency, which was low (<200 ug/g) in 9 patients (90%). Two of the 9 patients with low fecal elastase had concurrent ICI-related colitis, which may also lower fecal elastase values. Two patients who had follow-up testing 5 years after initial testing at CIADM diagnosis demonstrated a low fecal elastase, with 1 patient returning to normal range and the other having persisting low values in the range of exocrine insufficiency.

**Figure 4.**
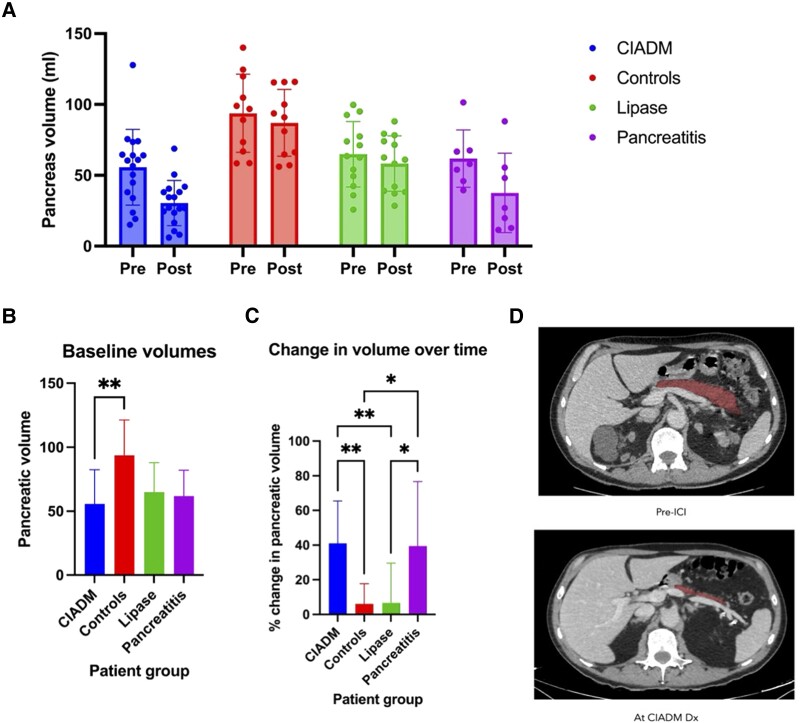
Pancreatic volumetry in ICI treated patients. (A) Pancreatic volumes for the 4 patient groups prior to ICI and post-diagnosis of CIADM/6 months of ICI use/lipase elevation/pancreatitis, respectively. Dots indicate individual values, columns indicates mean, and error bars indicate standard deviation. (B) Baseline pancreatic volumes for the 4 patient groups. (C) Percent change in volume over time for the 4 patient groups. (D) Representative CT slices from 1 patient taken at the level of the splenic vein. Pancreas highlighted in red. Abbreviations: CIADM, checkpoint inhibitor-associated autoimmune diabetes; CT, computed tomography; ICI, immune checkpoint inhibitor.

Baseline pancreatic volume was significantly different between the groups (Fig. 4B): CIADM group, ICI-treated control group, ICI-related lipase elevation group, and ICI-related pancreatitis group (F = 5.4, *P* = .003), with the CIADM group baseline pancreatic volume being significantly lower than the control group (*P* = .002). There was a progressive decline of 41% (mean) comparing the pretreatment to 6 months post-diagnosis of CIADM (Fig. 4C). An example of this progressive decline in pancreatic volume is portrayed in Fig. 4D with representative CT slices through the pancreas of a CIADM patient. This decline in the CIADM group is similar to the 39% decrease in pancreatic volume noted in the ICI-related pancreatitis cohort and significantly greater than the 6% reduction in ICI-treated controls and 6% decrement in those with isolated lipase elevation (F = 8.0, *P* < .001).

There was no significant difference between baseline pancreatic volumes of autoantibody positive and negative groups (*P* = .54) or HLA-susceptible and nonsusceptible groups (*P* = .18). There was also no significant difference between these groups when comparing percentage decline in pancreatic volume (*P* = .79 and *P* = .17 respectively).

## Follow-up

Oncological response was assessable in 32 patients, and 22 (69%) patients responded to ICI therapy (4 with complete response, 18 with partial response), 2 had stable disease, and 8 (25%) had progressive disease as best response. At a median follow-up of 35 months 13/22 responses were ongoing. 8/36 patients were deceased by time of data collection, and all deaths were due to disease progression. Of the 26 melanoma patients with oncological response reported, 20 (77%) responded (4 with complete response, 16 with partial response) and 6 had progressive disease as best response.

All of the CIADM patients remained insulin dependent on follow-up. All had low C-peptide at diagnosis or within 1 month (<0.4 nmol/L), and 6/9 had undetectable levels of C-peptide on ultrasensitive assay. Mean HbA1c on follow-up was 8.1% (range 5.9–10.1%). Thirty-two of 36 patients were managed with multiple daily insulin injections, and 4/36 used insulin pump therapy. Eight of 36 (22%) patients were deceased by time of follow-up due to melanoma.

## Discussion

The prevalence of autoantibodies in our cohort was 43%, and all antibody-positive patients were positive for antiglutamic acid decarboxylase antibodies. Given that the rate of autoantibody positivity in classic T1D patients is 90% (16), this significant discrepancy suggests that CIADM pathogenesis may be less contingent on humoral immunity or potentially may involve different epitopes in immune triggering. The survival curves in Fig. 1 showed a trend to earlier diagnosis in antibody-positive people that is consistent with our meta-analysis (11). In that series, we found that a serum C-peptide of <0.4 nmol/L on an ultrasensitive assay had 100% sensitivity in detecting patients with a long-term requirement for insulin, again consistent with our case series findings. We support the use of a rapid loss of C-peptide (<0.4 nmol/L by 1 month post-CIADM diagnosis) in conjunction with new-onset hyperglycemia (HbA1c ≥ 6.5% and/or blood glucose level ≥ 11 mmol/L) as diagnostic criteria for CIADM.

All patients in our CIADM cohort had exposure to either anti-PD-1 or anti-PD-L1 therapy, highlighting that inhibition of the PD-1/PD-L1 axis is critical to the pathogenesis of CIADM. This aligns with previously published cases of CIADM (5–9, 11, 17, 18). Published case reports of anti-CTLA-4 CIADM do not provide clear evidence of insulin deficiency or thorough exclusion of other forms of diabetes (18–20). The situation is reversed with checkpoint-inhibitor hypophysitis, where the rate of hypophysitis is higher with CTLA-4 therapy, alone or in combination with PD1 (21), suggesting important pathophysiological differences between the 2 irAEs.

The role of genetics appears different or potentially less important in CIADM in comparison to traditional T1D. Of the patients tested for HLA haplotypes, only 46% had a traditional T1D susceptibility haplotype (similar to 59.3% in the meta-analysis), and 15% in our case series and 7.6% in the meta-analysis had protective haplotypes (11). In comparison, among classic T1D patients 90% had a HLA susceptibility haplotype (14, 22). The lower prevalence of T1D susceptibility haplotypes in the CIADM population may reflect that, in surviving to the mean age of 66 years without T1D, the highest risk groups have already been selected out. Furthermore, the additional exposure of anti-PD1 in this context skews the balance of genetics vs environment greatly and may trigger other mechanisms of autoimmunity.

Pancreatic volumetry demonstrated rapid decline in pancreatic volume well beyond what would be expected with insulitis alone, which was consistent with other case series reporting pancreatic volumetry data in CIADM patients (12, 13). The degree of pancreatic volume loss was on par with those with overt ICI-related pancreatitis, while a lipase elevation in isolation did not lead to such significant volume loss. Interestingly, baseline pancreatic volume for CIADM patients was significantly lower compared with controls. This generates the hypothesis that CIADM patients have a lower pancreatic reserve and/or prior insult that puts them at increased risk of CIADM. Further studies with larger sample sizes will be needed to determine whether smaller pancreatic volume reliably predicts CIADM development.

This is the first study to assess and compare the extent of pancreatic volume loss between different pancreatic phenotypes including CIADM, pancreatitis, lipase elevation, and ICI-treated controls. Previously, it has been demonstrated both by radiologic analyses and by postmortem examination that pancreatic weight for patients with T1D is significantly lower than normal controls and even patients with type 2 diabetes, although it was not significantly affected by duration of diabetes (23, 24). Further analyses of pancreatic volume in patients with recent-onset T1D shows a 26% reduction in volume compared with healthy controls even at this early stage of disease (25), suggesting that reduction in pancreatic volume may not be a unique feature to CIADM but a feature of autoimmune diabetes progression. While ICI-related pancreatitis volume has not previously been characterized, it is known that chronic pancreatitis is associated with a pancreatic volume reduction of approximately 21% (26).

Combined with the relatively high incidence of elevated lipase at time of presentation (48%) and low fecal elastase values in 90% of those tested, exocrine pancreatic involvement seems to be a common feature in CIADM. Further follow-up data would be of interest to determine the extent to which exocrine function recovers if lost, as was the case in 1 of our patients with follow-up fecal elastase testing.

Our longitudinal lipase data is also the first to demonstrate a lipase rise that precedes the onset of diabetes, presumably signifying the onset of autoimmune exocrine pancreatic damage. Furthermore, treatment of patients on ICIs with an elevated lipase with steroids (as was done in 2 of these 4 patients) precipitated fulminant hyperglycaemia and DKA. Thus, CIADM should be closely monitored if treating people with elevated lipase with steroids. Moreover, CIADM was not prevented by steroids in these 2 patients.

Analysis of glucose values prior to formal CIADM diagnosis suggested that 40% of patients had abnormal glucose values preceding formal CIADM diagnosis. This indicates that improved clinical awareness could have led to earlier detection of CIADM and potential avoidance of DKA in these patients.

Our follow-up data on HbA1c values suggests that long-term glycemic management in these patients is challenging, and HbA1c targets are likely set relatively higher in view of older age, more comorbidities, and risks associated with hypoglycemia. Lower use of insulin pump therapy is due to limitations within health care systems and more limited capacity to facilitate the steep learning curve required.

## Conclusions

CIADM is an uncommon but highly morbid complication of ICI therapy that requires distinct diagnostic criteria from traditional T1D. Our results are consistent with CIADM co-occurring with significant pancreatic volume loss (with or without other features of pancreatic exocrine deficiency). Identifying the premorbid predictors of CIADM may allow targeted trials of treatments and earlier detection of this complication of ICI.

## Data Availability

Data from this study are available from the corresponding author on reasonable request.

## Abbreviations

CIADM: checkpoint inhibitor associated autoimmune diabetes
CT: computed tomography
CTLA-4: cytotoxic T-cell-associated antigen 4
DKA: diabetic ketoacidosis
HbA1c: glycosylated hemoglobin
HLA: human leukocyte antigens
ICI: immune checkpoint inhibitor
irAE: immune-related adverse effect
PD1: programmed cell death protein 1
PDL1: programmed cell death protein ligand 1
T1D: type 1 diabetes

## References

[1] Larkin J, Chiarion-Sileni V, Gonzalez R, et al Five-year survival with combined nivolumab and ipilimumab in advanced melanoma. N Engl J Med. 2019;381(16):1535‐1546. 31562797 10.1056/NEJMoa1910836

[2] Das S, Johnson DB. Immune-related adverse events and anti-tumor efficacy of immune checkpoint inhibitors. J Immunother Cancer. 2019;7(1):1‐11. 31730012 10.1186/s40425-019-0805-8 PMC6858629

[3] Scott ES, Long GV, Guminski A, Clifton-Bligh RJ, Menzies AM, Tsang VH. The spectrum, incidence, kinetics and management of endocrinopathies with immune checkpoint inhibitors for metastatic melanoma. Eur J Endocrinol. 2018;178(2):173‐180. 29187509 10.1530/EJE-17-0810

[4] Barroso-Sousa R, Barry WT, Garrido-Castro AC, et al Incidence of endocrine dysfunction following the use of different immune checkpoint inhibitor regimens a systematic review and meta-analysis. JAMA Oncol. 2018;4(2):173‐182. 28973656 10.1001/jamaoncol.2017.3064 PMC5838579

[5] Stamatouli AM, Quandt Z, Perdigoto AL, et al Collateral damage: insulin-dependent diabetes induced with checkpoint inhibitors. Diabetes. 2018;67(8):1471‐1480. 29937434 10.2337/dbi18-0002 PMC6054443

[6] Tsang VHM, McGrath RT, Clifton-Bligh RJ, et al Checkpoint inhibitor-associated autoimmune diabetes is distinct from type 1 diabetes. J Clin Endocrinol Metab. 2019;104(11):5499‐5506. 31265074 10.1210/jc.2019-00423

[7] De Filette JMK, Pen JJ, Decoster L, et al Immune checkpoint inhibitors and type 1 diabetes mellitus: a case report and systematic review. Eur J Endocrinol. 2019;181(3):363‐374. 31330498 10.1530/EJE-19-0291 PMC6709545

[8] Kotwal A, Haddox C, Block M, Kudva YC. Immune checkpoint inhibitors: an emerging cause of insulin-dependent diabetes. BMJ Open Diabetes Res Care. 2019;7(1):1‐10. 10.1136/bmjdrc-2018-000591 PMC6398813 30899528

[9] Yun K, Daniels G, Gold K, McCowen K, Patel SP. Rapid onset type 1 diabetes with anti-PD-1 directed therapy. Oncotarget. 2020;11(28):2740‐2746. 32733645 10.18632/oncotarget.27665 PMC7367652

[10] Haslam A, Prasad V. Estimation of the percentage of us patients with cancer who are eligible for and respond to checkpoint inhibitor immunotherapy drugs. JAMA Netw Open. 2019;2(5):1‐9. 10.1001/jamanetworkopen.2019.2535 PMC6503493 31050774

[11] Wu L, Tsang V, Menzies AM, et al Risk factors and characteristics of checkpoint inhibitor-associated autoimmune diabetes mellitus (CIADM): a systematic review and delineation from type 1 diabetes. Diabetes Care. 2023;46(6):1292‐1299. 37220262 10.2337/dc22-2202

[12] Byun DJ, Braunstein R, Flynn J, et al Immune checkpoint inhibitor– associated diabetes: a single-institution experience. Diabetes Care. 2020;43(12):3106‐3109. 33051330 10.2337/dc20-0609 PMC7770268

[13] Marchand L, Thivolet A, Dalle S, et al Diabetes mellitus induced by PD-1 and PD-L1 inhibitors: description of pancreatic endocrine and exocrine phenotype. Acta Diabetol. 2019;56(4):441‐448. 30284618 10.1007/s00592-018-1234-8

[14] Noble JA, Valdes AM. Genetics of the HLA region in the prediction of type 1 diabetes. Curr Diab Rep. 2011;11(6):533‐542. 21912932 10.1007/s11892-011-0223-x PMC3233362

[15] Djuric-Stefanovic A, Masulovic D, Kostic J, Randjic K, Saranovic D. CT volumetry of normal pancreas: correlation with the pancreatic diameters measurable by the cross-sectional imaging, and relationship with the gender, age, and body constitution. Surg Radiol Anat. 2012;34(9):811‐817. 22434256 10.1007/s00276-012-0962-7

[16] Bingley PJ . Clinical applications of diabetes antibody testing. J Clin Endocrinol Metab. 2010;95(1):25‐33. 19875480 10.1210/jc.2009-1365

[17] Chang LS, Barroso-Sousa R, Tolaney SM, Hodi FS, Kaiser UB, Min L. Endocrine toxicity of cancer immunotherapy targeting immune checkpoints. Endocr Rev. 2018;40(1):17‐65. 10.1210/er.2018-00006 PMC6270990 30184160

[18] Wright JJ, Salem JE, Johnson DB, et al Increased reporting of immune checkpoint inhibitor-associated diabetes. Diabetes Care. 2018;41(12):e150‐e151. 30305348 10.2337/dc18-1465 PMC7301161

[19] Liu J, Zhou H, Zhang Y, et al Reporting of immune checkpoint inhibitor therapy-associated diabetes, 2015-2019. Diabetes Care. 2020;43(7):E79‐E80. 32393586 10.2337/dc20-0459 PMC7305010

[20] Yamazaki N, Kiyohara Y, Uhara H, et al Phase II study of ipilimumab monotherapy in Japanese patients with advanced melanoma. Cancer Chemother Pharmacol. 2015;76(5):997‐1004. 26410424 10.1007/s00280-015-2873-x PMC4612321

[21] Arnaud-Coffin P, Maillet D, Gan HK, et al A systematic review of adverse events in randomized trials assessing immune checkpoint inhibitors. Int J Cancer. 2019;145(3):639‐648. 30653255 10.1002/ijc.32132

[22] Erlich H, Valdes AM, Noble J, et al HLA DR-DQ haplotypes and genotypes and type 1 diabetes risk: analysis of the type 1 diabetes genetics consortium families. Diabetes. 2008;57(4):1084‐1092. 18252895 10.2337/db07-1331 PMC4103420

[23] Campbell-Thompson ML, Filipp SL, Grajo JR, et al Relative pancreas volume is reduced in first-degree relatives of patients with type 1 diabetes. Diabetes Care. 2019;42(2):281‐287. 30552130 10.2337/dc18-1512 PMC6341284

[24] Campbell-Thompson ML, Kaddis JS, Wasserfall C, et al The influence of type 1 diabetes on pancreatic weight. Diabetologia. 2016;59(1):217‐221. 26358584 10.1007/s00125-015-3752-z PMC4670792

[25] Williams AJK, Thrower SL, Sequeiros IM, et al Pancreatic volume is reduced in adult patients with recently diagnosed type 1 diabetes. J Clin Endocrinol Metab. 2012;97(11):2109‐2113. 10.1210/jc.2012-1815 22879632

[26] Schrader H, Menge BA, Schneider S, et al Reduced pancreatic volume and β-cell area in patients with chronic pancreatitis. Gastroenterology. 2009;136(2):513‐522. 19041312 10.1053/j.gastro.2008.10.083

